# Supplementary material for: Enteric Pathogens in Wild Boars Across the European Union: Prevalence and Antimicrobial Resistance Within a One Health Framework
Source: Antibiotics (Basel). 2025 Dec 10;14(12):1246. doi: 10.3390/antibiotics14121246 (PMC12729840; doi:10.3390/antibiotics14121246)
Supplement: Supplementary file 1 [file antibiotics-14-01246-s001.zip › antibiotics-4004013-supplementary.pdf]

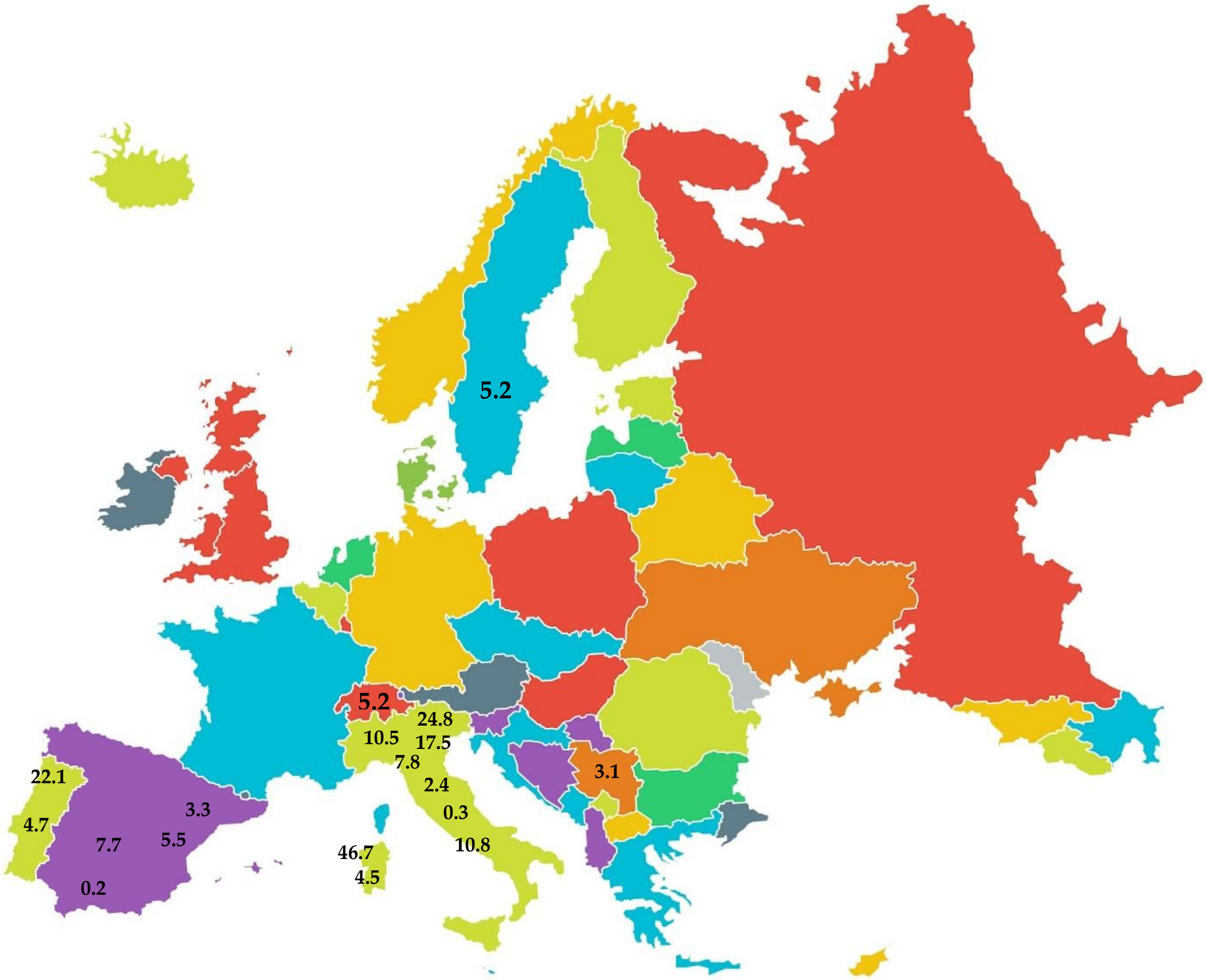

Figure S1. Prevalence (%) of *Salmonella* spp. wild boars carriers at faeces and/or lymph nodes and/or tonsil level in different countries of the European Union.

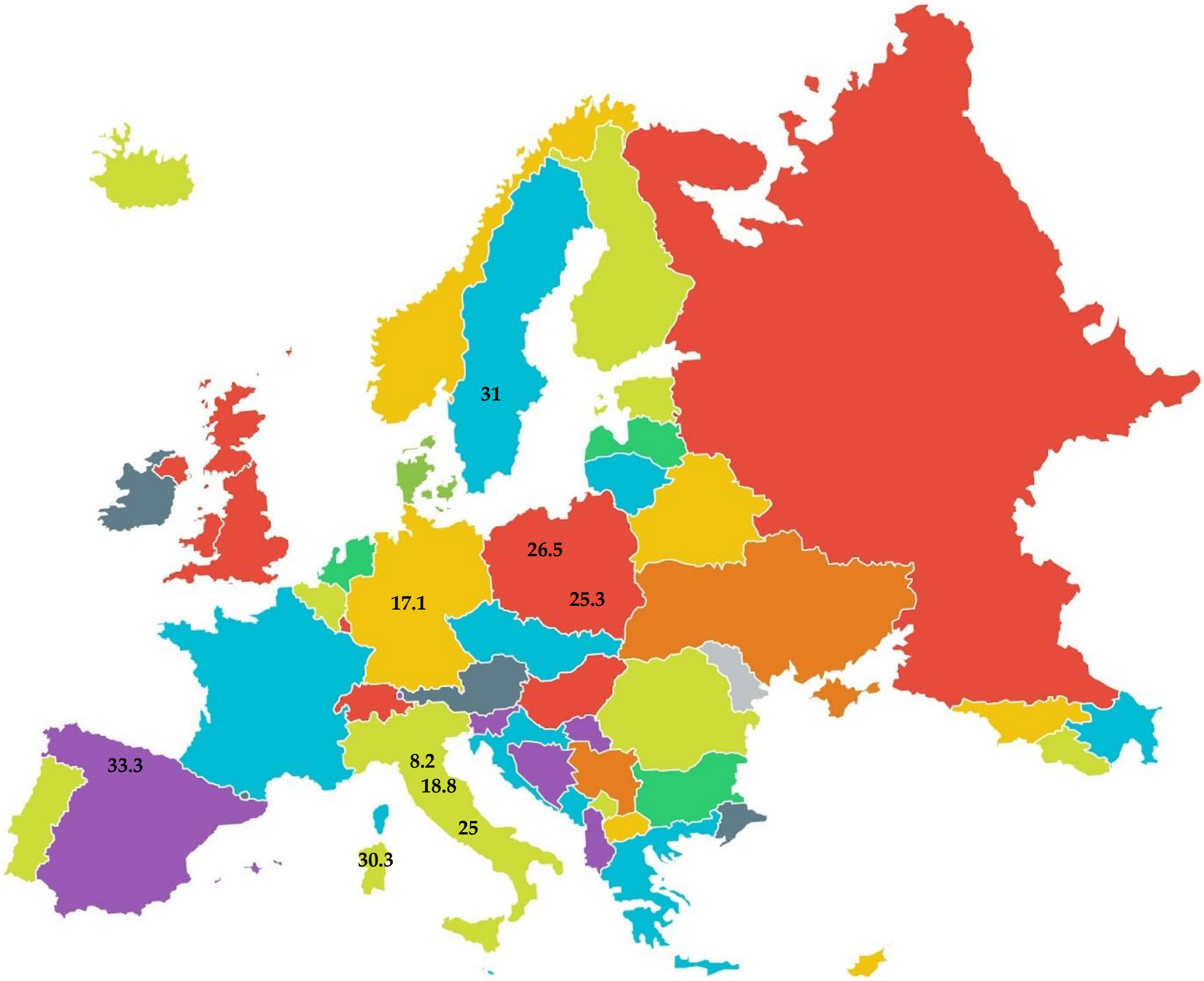

Figure S2. Prevalence (%) of *Y. enterocolitica* wild boars carriers at faeces and/or lymph nodes and/or tonsil level in different countries of the European Union.

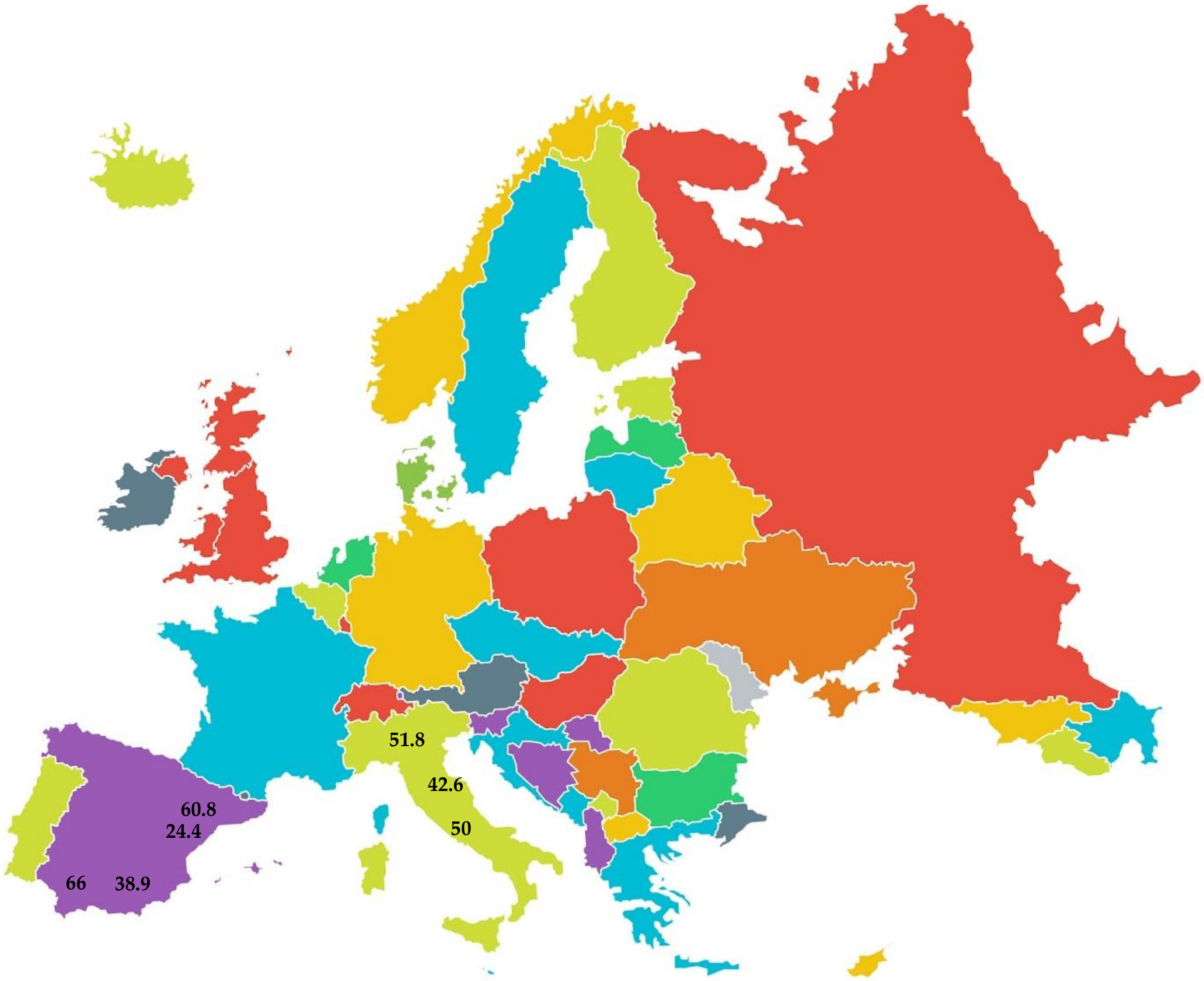

Figure S3. Prevalence (%) of *Campylobacter* spp. wild boars carriers at faeces level in different countries of the European Union.

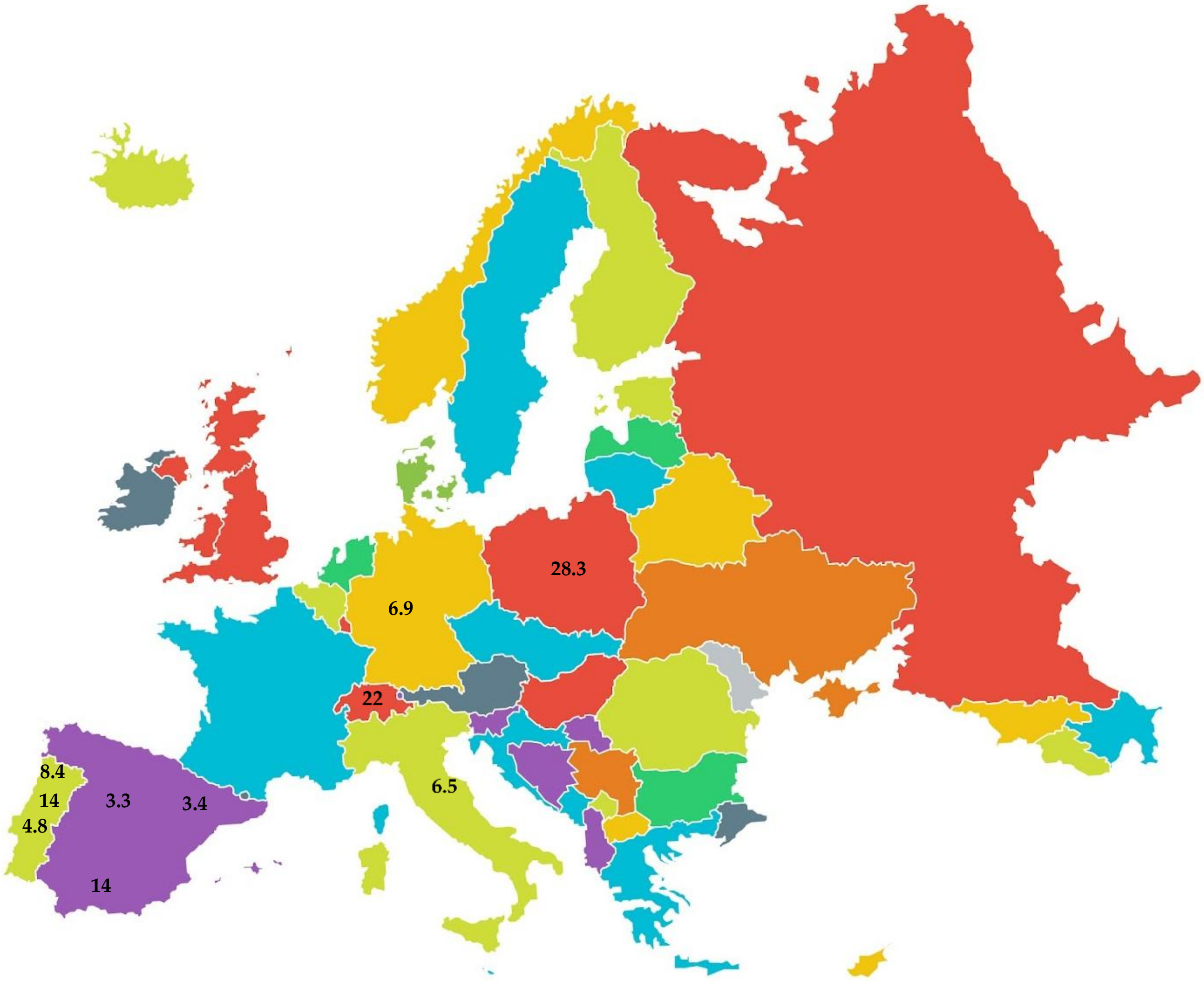

Figure S4. Prevalence (%) of *STEC* wild boars carriers at faeces level in different countries of the European Union.
